# Peripheral PD-1 and Tim-3 percentages are associated with primary sites and pathological types of peritoneal neoplasms

**DOI:** 10.1186/s12885-023-10752-2

**Published:** 2023-03-29

**Authors:** Huihui Hu, Jin Zhao, Judong Yuan, Man Zhang

**Affiliations:** 1 Department of Clinical Laboratory, Beijing Shijitan Hospital, Capital Medical University, 10 Tieyi Road, Haidian District, Beijing, 100038 China; 2 Beijing Key Laboratory of Urinary Cellular Molecular Diagnostics, Beijing, China; 3 Clinical Laboratory Medicine, Peking University Ninth School of Clinical Medicine, Beijing, 100038 China

**Keywords:** PD-1, Tim-3, Peritoneal neoplasms, Primary sites, Pathological types, Progression-free survival, Flow cytometry

## Abstract

**Purpose:**

Programmed death-1 (PD-1) and T cell immunoglobulin and mucin-domain-containing molecule 3(Tim-3) may be used as the biomarkers for the therapy in patients with peritoneal neoplasms. In the current study, the differential percentages of peripheral PD-1 and Tim-3 are explored to investigate whether to associate with primary sites and pathological types of patients with peritoneal neoplasms or not. We also investigated the frequencies of PD-1 and Tim-3 on circulating Lymphocytes, CD3 + T cells, CD3 + CD4 + T cells and CD3 + CD8 + T cells if would correlate with the progression-free survival of peritoneal neoplasms patients.

**Methods:**

115 patients with peritoneal neoplasms were recruited, subjected to multicolor flow cytometric analyses of the percentages of PD-1 and Tim-3 receptors of circulating Lymphocytes, CD3 + T cells, CD3 + CD4 + T cells and CD3 + CD8 + T cells. The peritoneal neoplasms patients were divided into primary group and secondary group depending on whether the tumor had primary focus and limited to peritoneal tumor or not. Then all the patients were regrouped by the pathological types of neoplasms (adenocarcinoma, mesothelioma, and pseudomyxoma). The secondary peritoneal neoplasms group was divided into the different primary site groups (colon, gastric, gynecology). This study also enrolled 38 cases of normal volunteers. The above markers were explored by flow cytometer, to find the differential levels in peritoneal neoplasms patients compared with normal group in peripheral blood.

**Results:**

Higher levels of CD4 + T lymphocytes, CD8 + T lymphocytes, CD45 + PD-1 + lymphocytes, CD3 + PD-1 + T cells, CD3 + CD4 + PD-1 + T cells, CD3 + CD8 + PD-1 + T cells and CD45 + Tim-3 + lymphocytes were found in peritoneal neoplasms group than normal control (the p value was respectively 0.004, 0.047, 0.046, 0.044, 0.014, 0.038 and 0.017). Compared with primary peritoneal neoplasms group, the percentages of CD45 + PD-1 + lymphocytes, CD3 + PD-1 + T cells, and CD3 + CD4 + PD-1 + T cells were increased in the secondary peritoneal neoplasms group (the p value was respectively 0.010, 0.044, and 0.040), while PD-1 did not correlate with the primary sites in secondary group (P > 0.05). Tim-3 had no statistical differences in primary peritoneal neoplasms group compared with secondary group (p > 0.05), but CD45 + Tim-3+% lymphocytes, CD3 + Tim-3+%T cells, and CD3 + CD4 + Tim-3 + T cells were associated with different secondary sites of peritoneal neoplasms (p < 0.05). In the different pathological type groups, the percentages of CD45 + PD-1 + lymphocytes, CD3 + PD-1 + T cells presented the higher levels in adenocarcinoma group compared with mesothelioma group (p = 0.048, p = 0.045). The frequencies of CD45 + PD-1 + lymphocytes and CD3 + PD-1 + T cells in peripheral blood were associated with progression-free survival (PFS).

**Conclusions:**

Our work uncovers peripheral PD-1 and Tim-3 percentages are associated with primary sites and pathological types of peritoneal neoplasms. Those findings might provide important assessment to predict peritoneal neoplasms patients’ immunotherapy responses.

## Introduction

Programmed cell death receptor-1(PD-1) and T cell immunoglobulin mucin-3 (Tim-3) are considered to be the popular molecules of immunosuppression in tumor immune escape and progression. PD-1 belongs to the member of the B7/CD28 family [1], various studies have demonstrated PD-1 was expressed in activated B cells, CD4 + T cells, CD8 + T cells, and NK T cells [2, 3]. Evidences show that blocking the immune checkpoint of PD-1 has been applied in the therapy of many human cancers clinically and experimentally, such as melanoma, non-small-cell lung cell cancer, colorectal cancer, and bladder cancer [4–7]. Since PD-1 is closely correlated with the dysfunctions of CD4 + T cells and CD8 + T cells, the clinical research of the antibody against PD-1 is effective. However, according to the reports, many patients with cancers are not sensitive to the blockade of PD-1 [8], so it is indispensable to discover the novel immune checkpoints that could be applied to provide more efficacious treatments. T cell immunoglobulin and mucin-domain-containing molecule 3(Tim-3) is a member of TIM family and is generally perceived as a checkpoint receptor. Researches show Tim-3 could appear as the co-inhibitory receptor. According to the reports, Tim-3 is expressed on IFN-γ producing T cells, Treg cells, macrophages and dendritic cells [9, 10]. Animal experiments demonstrate the blockade of Tim-3 can increase IFN-γ secretion [11]. Recent studies suggest PD-1 and Tim-3 are co-expressed during exhausted T cell differentiation, their crosstalk plays vital role in regulating T cell exhaustion and immunotherapy efficacy [8].

Peritoneal neoplasms are becoming the common malignant tumors in human recent years. The most prominent feature of peritoneal neoplasms is the insidious onset, that let these tumors become difficult to diagnose and treat [12]. Therefore, it is of great clinical significance to find an effective treatment method. Most peritoneal neoplasms are malignant metastatic tumors of the abdomen, and some are primary tumors (the tumors were confined to the peritoneum or abdominal cavity, and no primary lesions were found), including pseudomyxoma, myxoma, mesothelioma. Most peritoneal neoplasms are metastases of tumors from other organs in the body. This type of the tumors is called secondary peritoneal neoplasms. The primary sites of secondary peritoneal neoplasms always come from the colon, gastric, uterus, oviduct and other abdominal organs. The treatments of the secondary neoplasms, that come from the peritoneal metastases originating from gastrointestinal malignancies and gynecological malignancies have made continuous progress in the past 30 years. The treatments include cytoreductive surgery (CRS) and hyperthermic intraperitoneal chemotherapy (HIPEC). Chemotherapies for peritoneal neoplasms mainly include perioperative chemotherapy and regional chemotherapy. The side effects of chemotherapy are often great. Immune checkpoint blockade with anti-PD-1 and Tim-3 antibodies have become a hot topic for the treatment of human malignancies [13–15]. However, the application of immunosuppressive therapy in peritoneal neoplasms is rarely reported, and it remains unclear how PD-1 and Tim-3 impact on the prognosis of peritoneal neoplasms.

Therefore, in the present study, we explored peripheral PD-1 and Tim-3 expressed on circulating lymphocytes, CD3 + T cells, CD3 + CD4 + T cells and CD3 + CD8 + T cells whether to associate with primary sites and pathological types of peritoneal neoplasms or not. The T cell levels of PD-1 and Tim-3 assumed to induce intratumorally immune tolerance. Bekos et al. had reported in the tissue level, the percentages of CD8, PD-1, and PD-L1 expressed on tumor infiltrating leucocytes, had differential expressions between primary ovarian tissues and metastases intraperitoneal implants [16]. Due to the peripheral blood analyses were easier to perform, the focus of this article was to explore the differential percentages of PD-1 and Tim-3 exhibited on circulating lymphocytes of secondary peritoneal neoplasms group, compared with primary group. Heterogeneity was a characteristic of malignant tumors, in this light, we also investigated whether peripheral PD-1 and Tim-3 correlated with pathological types of peritoneal neoplasms (adenocarcinoma, mesothelioma, and pseudomyxoma). We hypothesized that peripheral blood analyses of PD-1 and Tim-3 expressed on lymphocytes, CD3 + T cells, CD3 + CD4 + T cells and CD3 + CD8 + T cells would provide new insights about the mechanism of peritoneal neoplasms progression.

## Patients and methods

### Patients

The peritoneal neoplasms patients were approached for enrollment between April 2021 and May 2022 from Beijing Shijitan Hospital, the affiliated cancer hospital of Capital Medical University. All 115 included patients received no previous PD-1 and other immunosuppressive agents checkpoint blockade. Peripheral blood samples were obtained from patients in clinical laboratory. Peripheral blood samples were also collected from 38 healthy gender- and age-matched controls. The peritoneal neoplasms patients were divided into primary group and secondary group depending on whether the tumor had primary focus and limited to peritoneal tumor or not. Because the sample size is limited, then all the patients were regrouped by the pathological types of neoplasms (adenocarcinoma, mesothelioma, and pseudomyxoma). The secondary peritoneal neoplasms group was divided into the different primary site group (colon, gastric, gynecology). The patients’ details were listed in Table 1. This study had gained approval from the institutional review board for the protection of human subjects, and all participants gave informed consent.

**Table 1:** Characteristics of peritoneal neoplasms patients

Group	Number(n)	Age(y)	Gender(F/M)
Primary	23	56.7 ± 11.6	16/7
Secondary Primary sites Colon Ovary Gastric Liver Oviduct Epityphlon Uterus Other	92 31 17 11 4 3 8 5 13	55.4 ± 9.0 54.5 ± 10.1 57.6 ± 9.5 50.9 ± 7.5 52.6 ± 11.5 55.6 ± 7.8 58.6 ± 9.6 54.5 ± 8.5 58.6 ± 10.1	62/30 18/13 17/0 6/5 2/2 3/0 5/5 5/0 6/5
Pathological types Adenocarcinoma Mesothelioma Pseudomyxoma Myxoma Clear-cell carcinoma Squamous-cell carcinoma Leiomyosarcoma Other	70 10 7 3 5 3 2 15	55.2 ± 13.1 60.4 ± 8.3 54.6 ± 9.5 56.8 ± 10.2 58.6 ± 9.3 53.6 ± 10.5 58.6 ± 9.3 57.5 ± 8.7	57/13 2/8 4/3 1/2 2/3 1/2 1/1 10/5

Age is presented as mean ± SD

### Flow cytometry

Beckman Coulter ten color flow cytometer (navios, USA) was used for this experiment. Before the experiment, the optical path and voltage conditions of the instrument were controlled. Fresh venous blood samples were collected from patients with EDTA-coated vacutainer tubes. Specific antibodies against PE anti-human CD3 (Biolegend, 300,308, USA), PerCP/Cyanine5.5 anti-human CD366 (Tim-3) (Biolegend, 345,016, USA), APC anti-human CD279 (PD-1) (Biolegend, 329,908, USA), APC/Fire™ 750 anti-human CD4(Biolegend, 300,560, USA), Pacific Blue™ anti-human CD8(Biolegend, 344,718, USA), Brilliant Violet 510™ anti-human CD45(Biolegend, 304,036, USA) were used. Fresh venous blood samples labeled with corresponding fluorochrome conjugated non-immune isotypes were taken as negative controls. The violet amine reactive dye (Invitrogen, USA) was used to assess the viability of the cells. After compensation setup, 200ul of fresh venous blood sample was mixed with 5ul of each antibody, vortex at high speed for 6–8 s and incubate for 15 min at room temperature. After incubation, each tube was mixed with 2ml of erythrolysin (Beckman Coulter, 200,072, USA), and incubate for 15 min at room temperature. Perform wash steps with 3ml 1XPBS each; centrifuge at 210 g for 5 min; aspirate the supernatant and re-suspend in 500ul of 1XPBS (containing 0.1% formaldehyde) and acquired on the flow cytometer. Before detection, the flow cytometer volt and fluorescence compensation values were set. For the analyses of PD-1 and Tim-3 on the surface of lymphocytes, CD3 + T cells, CD3 + CD4 + T cells and CD3 + CD8 + T cells, 5000 events were detected for each sample. And last, all the data were analyzed by Kaluza software (Beckman, USA).

### Statistical data analysis

The data were analyzed by GraphPad Prism 5.0 software (GraphPad Software, USA). The percentages of positive target cells in peripheral blood were used to compare the differences. The data were expressed by median. Comparison between the two groups was performed by independent sample T test or Mann-Whitney U nonparametric test to compute the P value. Analysis of variance (one-way ANOVA) was used to compare the three groups with Bonferroni correction. Bilateral P < 0.05 indicated significant difference.

### Ethics approval

This study was approved by Medical Ethics Committee, Beijing Shi Ji Tan Hospital, Capital Medical University, and was performed in accordance with the ethical standards as laid down in the Declaration of Helsinki. The informed consents were obtained from all participants.

## Results

### The differential percentages of CD3 + CD4 + T cells, CD3 + CD8 + T cells, and PD-1& Tim-3 positive T cells in peritoneal neoplasms patients compared with normal control

**Fig. 1:**
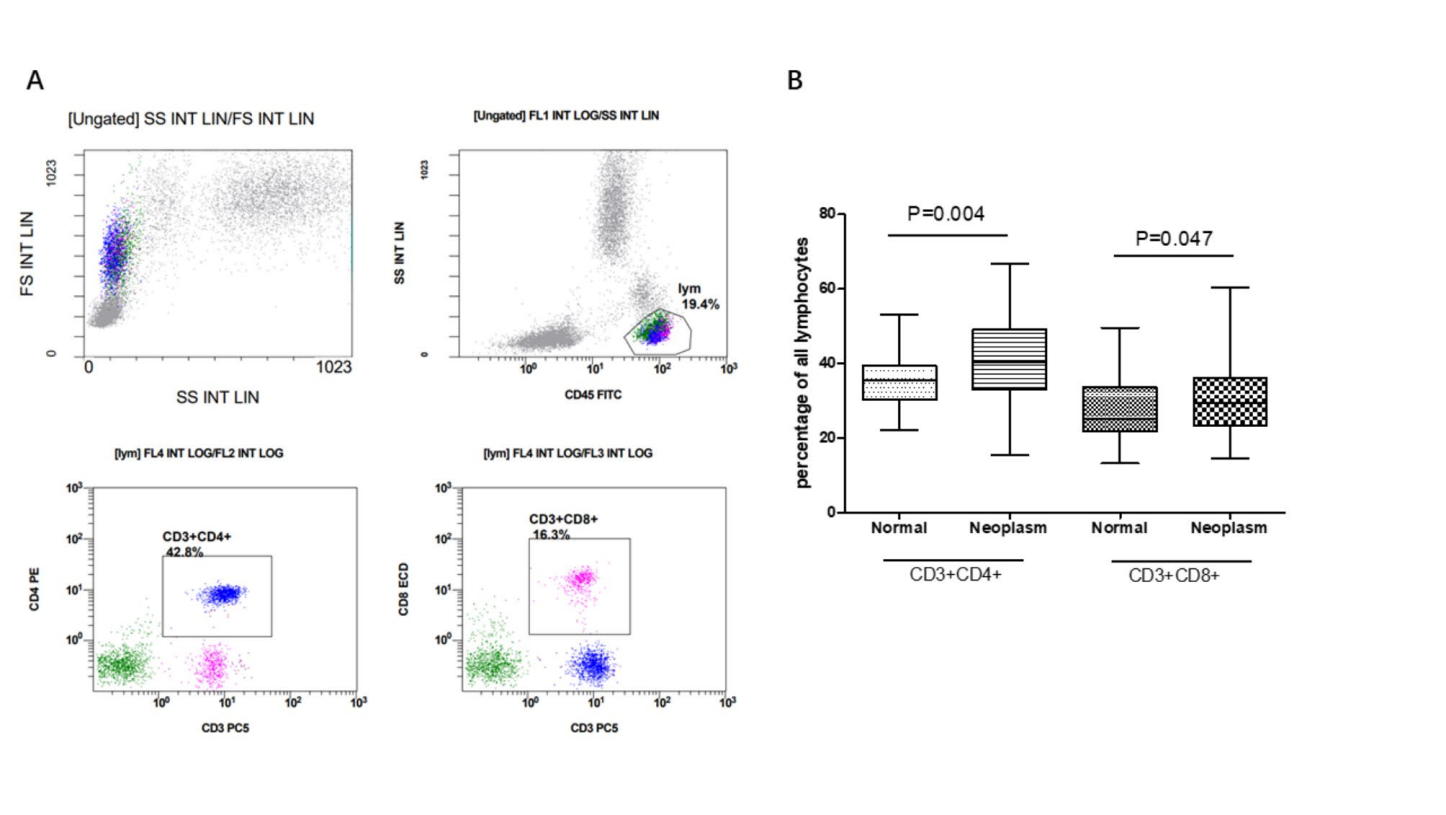
The differential levels of CD4 + T lymphocytes and CD8 + T lymphocytes between peritoneal neoplasms patients and normal control. (A: The differential percentages of CD4 + T lymphocytes and CD8 + T lymphocytes by flow cytometry; B: The statistical analysis of CD4 + T lymphocytes and CD8 + T lymphocytes between peritoneal neoplasms and normal group; P < 0.05 was considered as statistical significance)

**Fig. 2:**
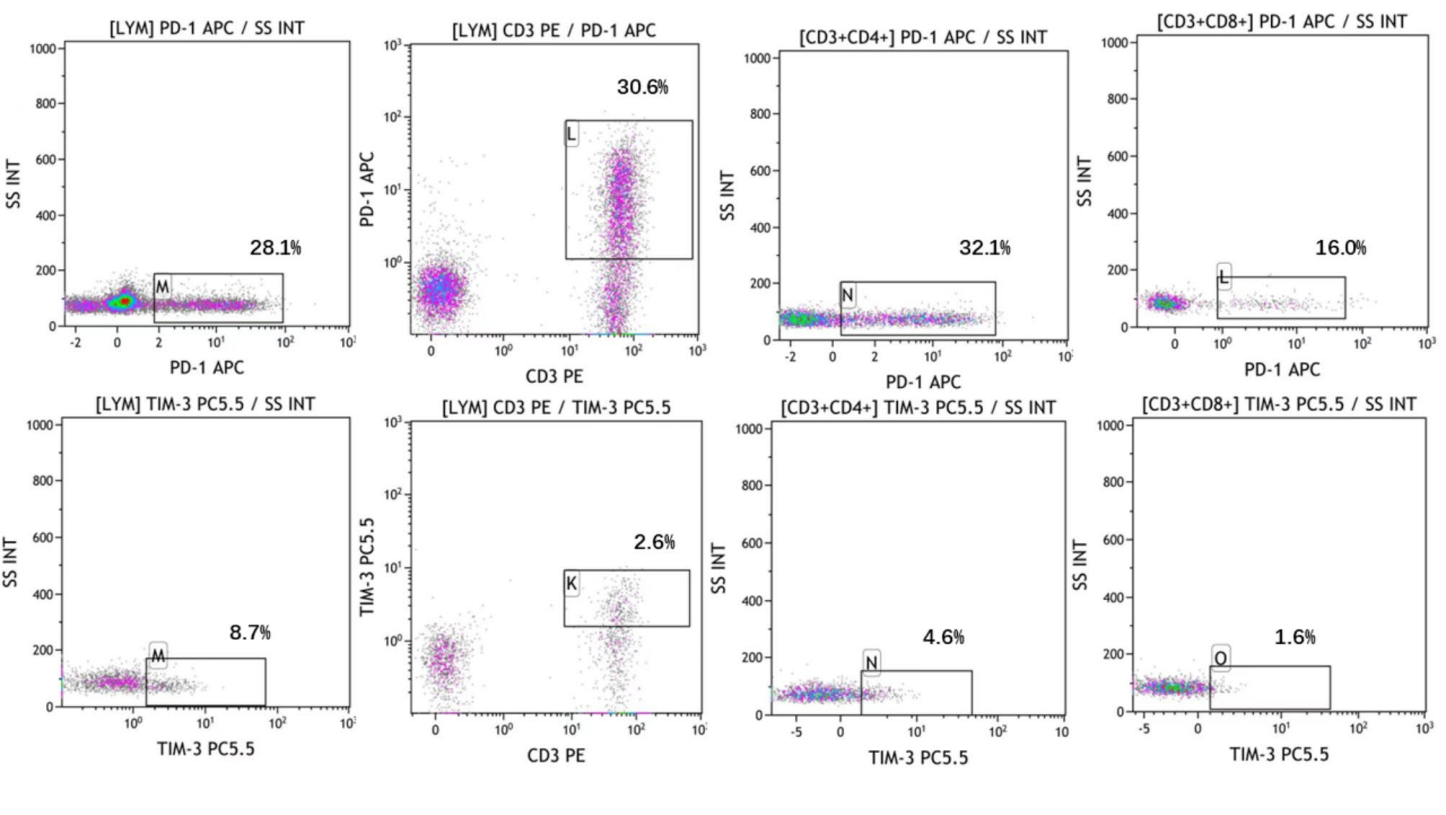
The differential percentages of PD-1 and Tim-3 on circulating lymphocytes, CD3 + T cells, CD3 + CD4 + T cells and CD3 + CD8 + T by flow cytometry

**Fig. 3:**
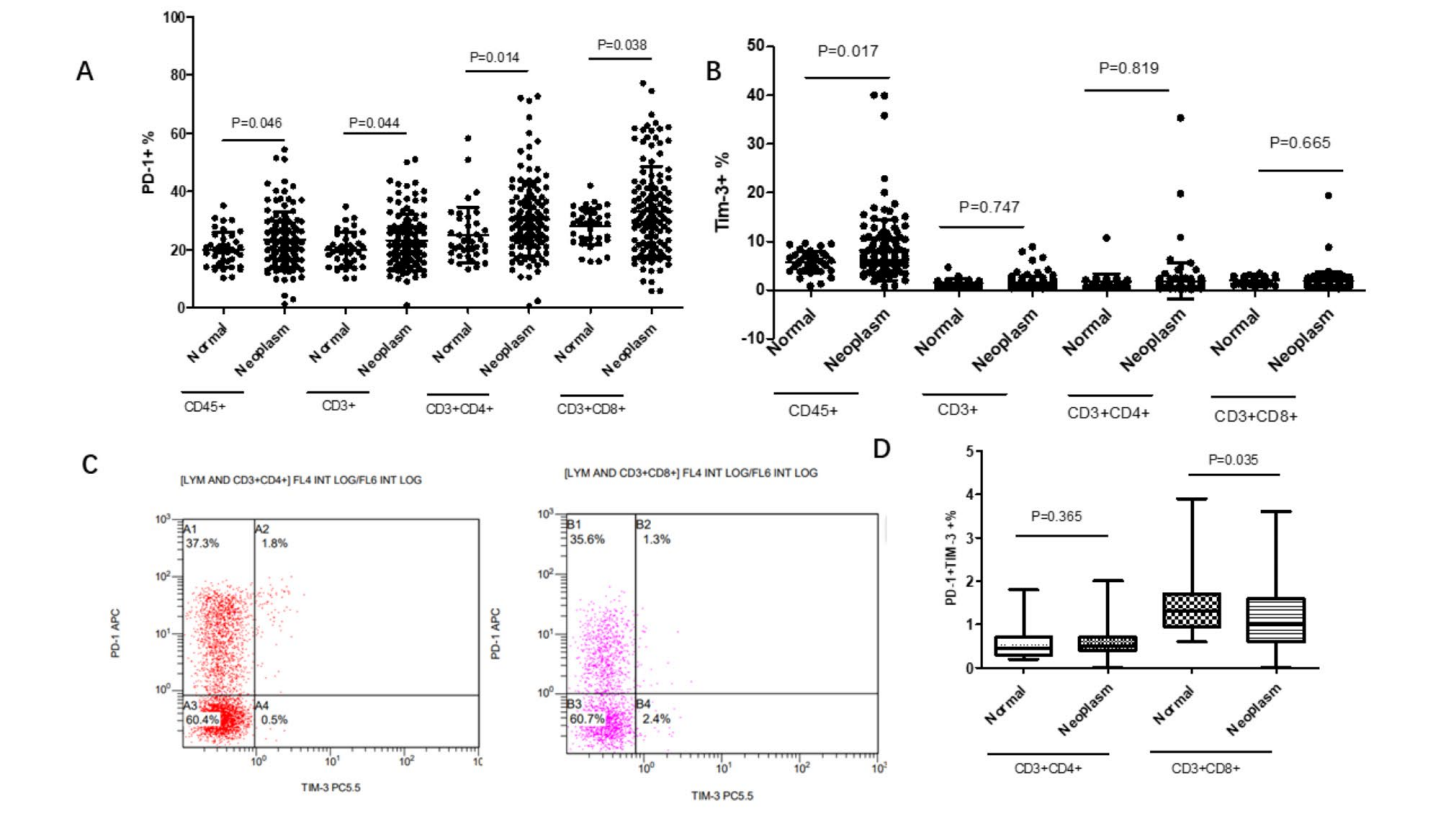
The levels of PD-1 and Tim-3 on circulating lymphocytes, CD3 + T cells, CD3 + CD4 + cells, and CD3 + CD8 + T cells between peritoneal neoplasms patients and normal control. (A: The statistical analyse of PD-1 + exhibited on lymphocytes, CD3 + T cells, CD3 + CD4 + cells, and CD3 + CD8 + T cells; B: The statistical analysis of Tim-3 + exhibited on lymphocyte, CD3 + T cells, CD3 + CD4 + cells, and CD3 + CD8 + T cells; C: The differential percentages of PD-1 + Tim-3 + CD4 + T lymphocytes and PD-1 + Tim-3 + CD8 + T lymphocytes by flow cytometry; D: The statistical analysis of PD-1 + Tim-3 + CD4 + T lymphocytes and PD-1 + Tim-3 + CD8 + T lymphocytes between peritoneal neoplasms patients and normal control; P < 0.05 was considered as statistical significance)

As shown in the Fig. 1, compared with normal control, the percentages of CD3 + CD4 + T cells and CD3 + CD8 + T cells were increased in the peripheral blood with peritoneal neoplasms patients (p = 0.004, p = 0.047, Fig. 1B). We also investigated whether the percentages of PD-1 and Tim-3 on circulating T lymphocytes had any differences between peritoneal neoplasms patients and normal volunteers or not (Fig. 2). Flow diagrams of PD-1 and Tim-3 exhibited on circulating lymphocyte, CD3 + T cells, CD3 + CD4 + T cells and CD3 + CD8 + T cells were shown in Fig. 3.The elevated percentages of CD45 + PD-1 + lymphocytes, CD3 + PD-1 + T cells, CD3 + CD4 + PD-1 T cells and CD3 + CD8 + PD-1T cells in peripheral blood were significantly higher than normal control (the p value was respectively 0.046, 0.044, 0.014, and 0.038, Fig. 2A).

CD45 + Tim-3 + lymphocytes were also exhibited at elevated levels in peritoneal neoplasms patients’ peripheral blood (p = 0.017, Fig. 2B). Unlike PD-1, the differential frequencies of CD3 + Tim-3 + T cells, CD3 + CD4 + Tim-3 T cells and CD3 + CD8 + Tim-3 T cells in peripheral blood between above groups had no statistical significances (the p value was respectively 0.747, 0.819, and 0.665, Fig. 2B). However, we also explored the frequencies of CD3 + CD4 + PD-1 + Tim-3 + T cells and CD3 + CD8 + PD-1 + Tim-3 + T cells between neoplasms group and normal control (Fig. 2C). There were no differences in CD3 + CD4 + PD-1 + Tim-3 + T cells percentages between above two groups. CD3 + CD8 + PD-1 + Tim-3 + T cells in control group were significantly higher than peritoneal neoplasms patients’ peripheral blood. This was contrary to the differential levels of CD3 + CD8 + PD-1 + T cells and CD3 + CD8 + Tim-3 + T cells in the two groups (Fig. 2D).

### Higher PD-1 levels on circulating lymphocytes in secondary peritoneal neoplasms patients compared with primary group

**Fig. 4:**
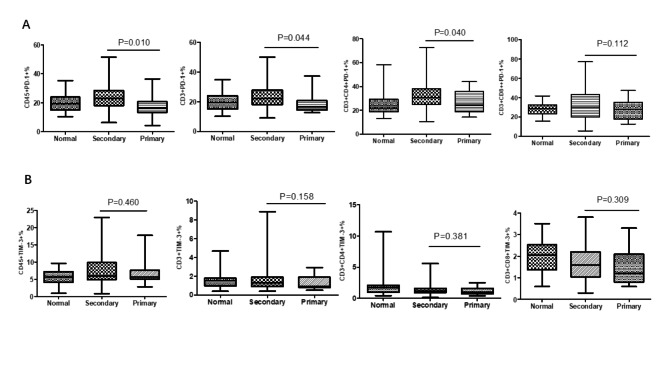
The levels of PD-1 and Tim-3 on circulating lymphocyte, CD3 + T cells, CD3 + CD4 + T and CD3 + CD8 + T cells between primary peritoneal neoplasms group and secondary group. (A: PD-1 presented the higher levels in secondary group compared with primary group; B: The levels of Tim-3 between secondary group and primary group had no statistical differences; P < 0.05 was considered as statistical significance.)

We compared the differential percentages of PD-1 and Tim-3 on circulating lymphocytes, CD3 + T cells, CD3 + CD4 + T cells and CD3 + CD8 + T cells between primary peritoneal neoplasms group compared with secondary group by flow cytometry.(Fig. 4). Compared with primary peritoneal neoplasms group, the percentages of CD45 + PD-1+, CD3 + PD-1+, and CD3 + CD4 + PD-1 + were increased in the secondary peritoneal neoplasms group (the p value was respectively 0.010, 0.044, and 0.040, Fig. 4A), while PD-1 expressed on CD3 + CD8 + T cells had no difference (p = 0.112, Fig. 4A). At the same time, the percentages of CD45 + Tim-3 + lymphocytes, CD3 + Tim-3 + T cells, CD3 + CD4 + Tim-3 + T cells, and CD3 + CD8 + Tim-3 + T cells had no statistical differences in primary peritoneal neoplasms group compared with secondary group (the p value was respectively 0.460, 0.158, 0.381, and 0.309, Fig. 4B).

### Differential percentages of Tim-3 were found on circulating lymphocytes in secondary peritoneal neoplasms group

**Fig. 5:**
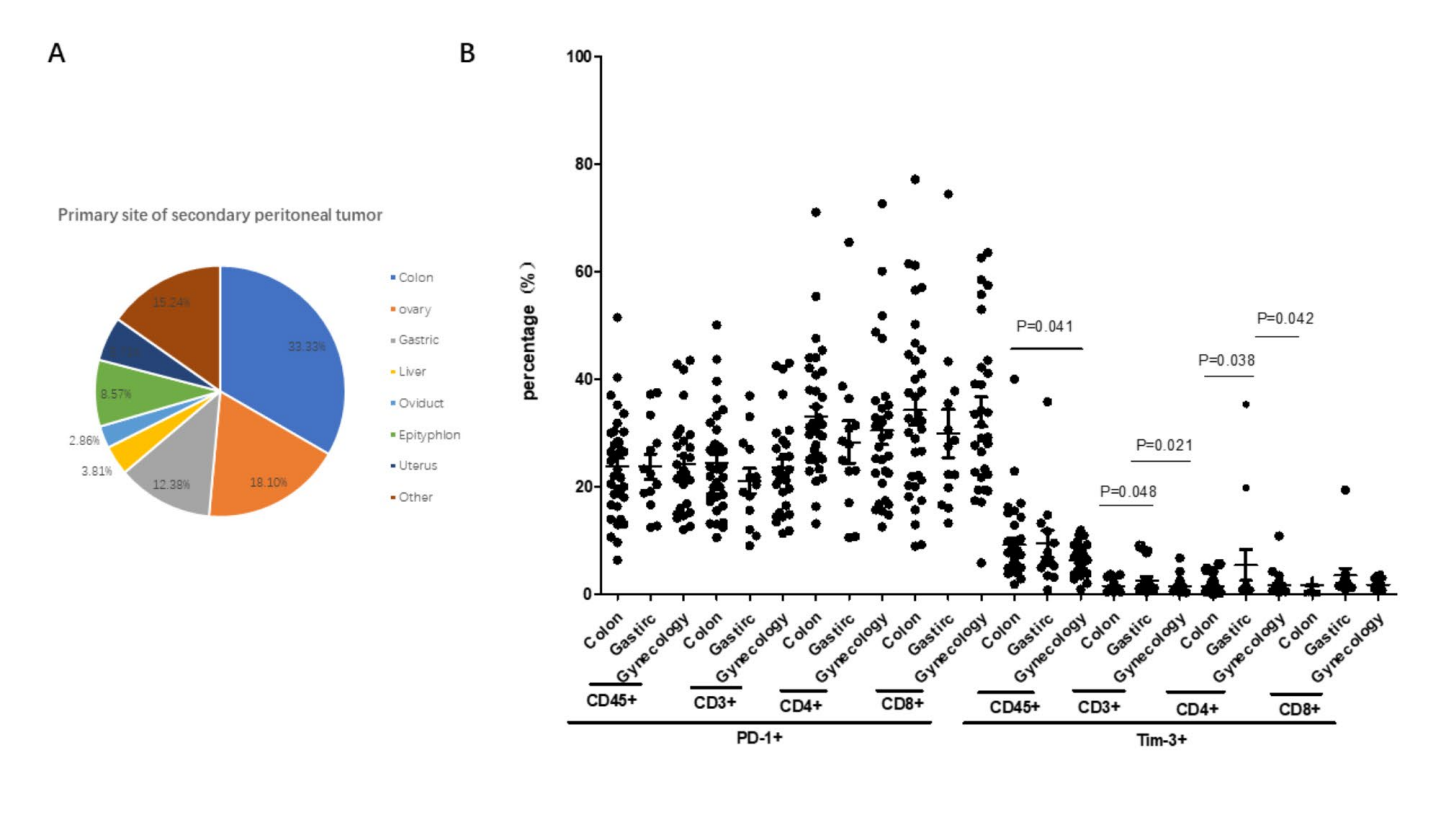
Differential percentages of PD-1 and Tim-3 were explored on circulating lymphocytes whether correlated with primary sites of secondary peritoneal neoplasms group. (A: Percentages of different primary sites; B: Compared with PD-1, the correlation between Tim-3 and the primary sites had statistical differences.)

The secondary peritoneal neoplasms group was divided into the different primary site groups (colon, ovary, gastric, liver, oviduct, epityphlon, uterus). Figure 5 A showed the proportion of primary sites of secondary peritoneal neoplasms we collected. Considering the sample size, we combined ovary, oviduct, and uterus in gynecology group. Then the differential percentages of PD-1 and Tim-3 were explored in colon, gastric and gynecology secondary peritoneal neoplasms groups. The percentages of CD45 + PD-1 + lymphocytes, CD3 + PD-1 + T cells, CD3 + CD4 + PD-1 + T cells in peripheral blood were not correlated with the different primary sites in secondary peritoneal neoplasms group (p > 0.05, Fig. 5B). Compared with PD-1, Tim-3 had lower percentages in peritoneal neoplasms group. While differential percentages of Tim-3 were found on circulating lymphocytes in secondary peritoneal neoplasms group. Compared with gynecology group, the percentages of CD45 + Tim-3 + lymphocytes were increased in colon group (6.26% vs. 9.14%; p = 0.041, Fig. 5B). CD3 + Tim-3 + T cells were also expressed at elevated levels in gastric secondary peritoneal neoplasm patients’ peripheral blood, respectively compared with colon group and gynecology group (2.52% vs. 1.50%; p = 0.048; 2.52% vs. 1.22%; p = 0.021, Fig. 5B). The above differences also appeared on circulating CD3 + CD4 + Tim-3 + T cells. CD3 + CD4 + Tim-3 + T cells had higher frequencies in gastric secondary peritoneal neoplasms patients’ peripheral blood, respectively compared with colon group and gynecology group (5.38% vs. 1.54%; p = 0.038; 5.38% vs. 1.64%; p = 0.042, Fig. 5B). The levels of PD-1 and Tim-3 exhibited on the CD3 + CD8 + T cells did not associate with primary sites of secondary peritoneal neoplasms group (p > 0.05, Fig. 5B).

### Compared with Tim-3, percentages of PD-1 correlated with pathological types of peritoneal neoplasms

**Fig. 6:**
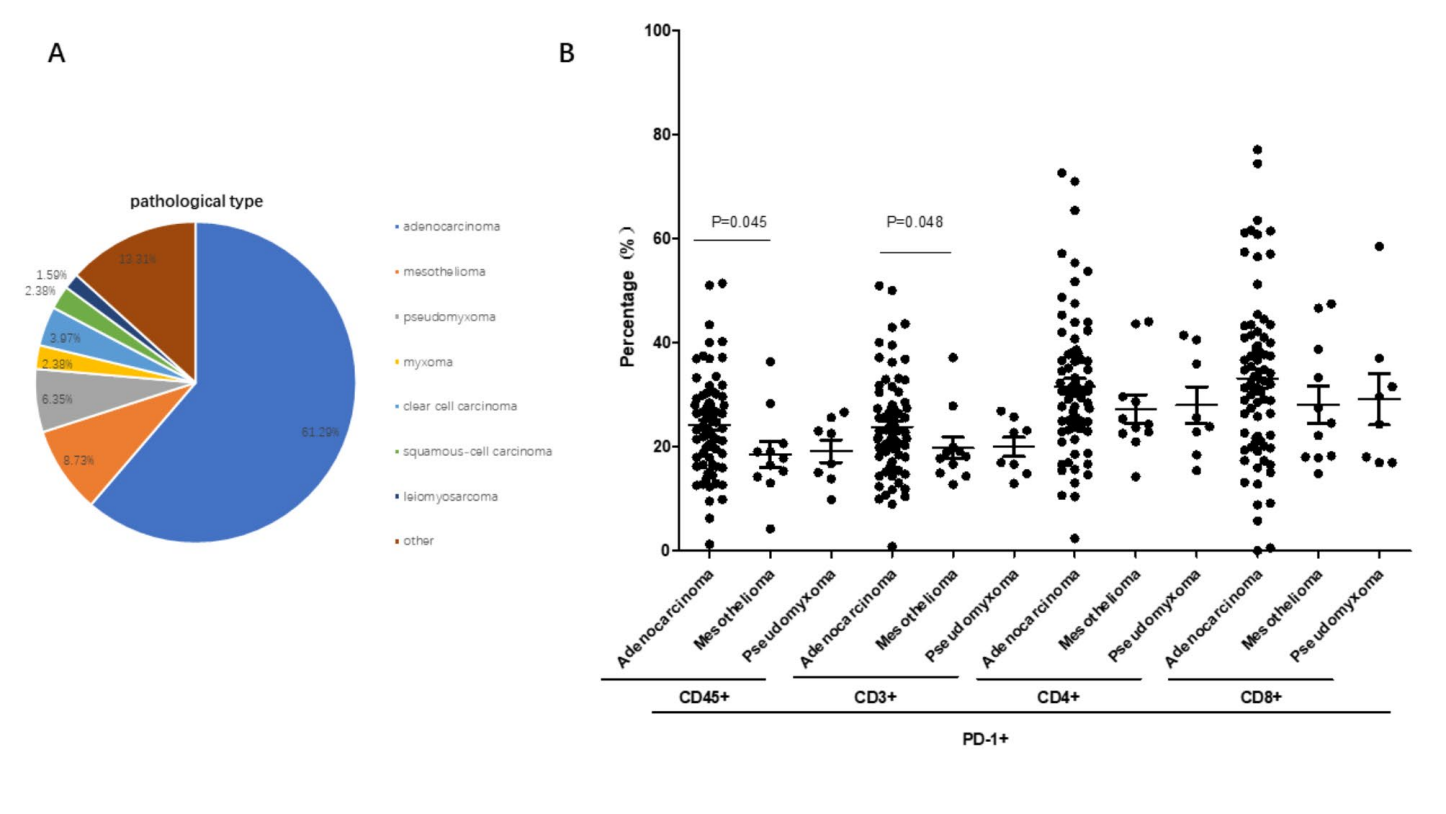
The percentages of PD-1 correlated with pathological types of peritoneal neoplasms. (A: Percentages of different pathological types of peritoneal neoplasms; B: The levels of PD-1 presented on circulating lymphocyte, CD3 + T cells, CD3 + CD4 + and CD3 + CD8 + T cells)

According to the different types of pathological tissue, the peritoneal neoplasms patients were divided into the following groups: adenocarcinoma, mesothelioma, pseudomyxoma, myxoma, clear cell carcinoma, squamous-cell carcinoma, leiomyosarcoma. Figure 6 A showed the proportion of pathology types of peritoneal neoplasms we collected. Because the sample size is limited, the differential frequencies of PD-1 and Tim-3 were explored in adenocarcinoma, mesothelioma, and pseudomyxoma. The frequencies of Tim-3 on circulating lymphocytes had no differences in peripheral blood among above groups.

We then asked whether the percentages of PD-1 had any differences on adenocarcinoma, mesothelioma, and pseudomyxoma groups. Compared with mesothelioma group, in the peripheral blood, higher percentages of circulating CD45 + PD-1 + lymphocytes, (18.62% vs. 24.21%; p = 0.045) and CD3 + PD-1 + T cells (19.83% vs. 23.83%; p = 0.048) were found in adenocarcinoma group (Fig. 6B). Because of the sample size is limited, the above differences were not obvious. No statistical differences were discovered in the percentages of circulating CD3 + CD4 + PD-1 + T cells and CD3 + CD8 + PD-1 + T cells (p > 0.05, Fig. 6B).

### PD-1 and Tim-3 vs. progression-free survival (PFS)

**Fig. 7:**
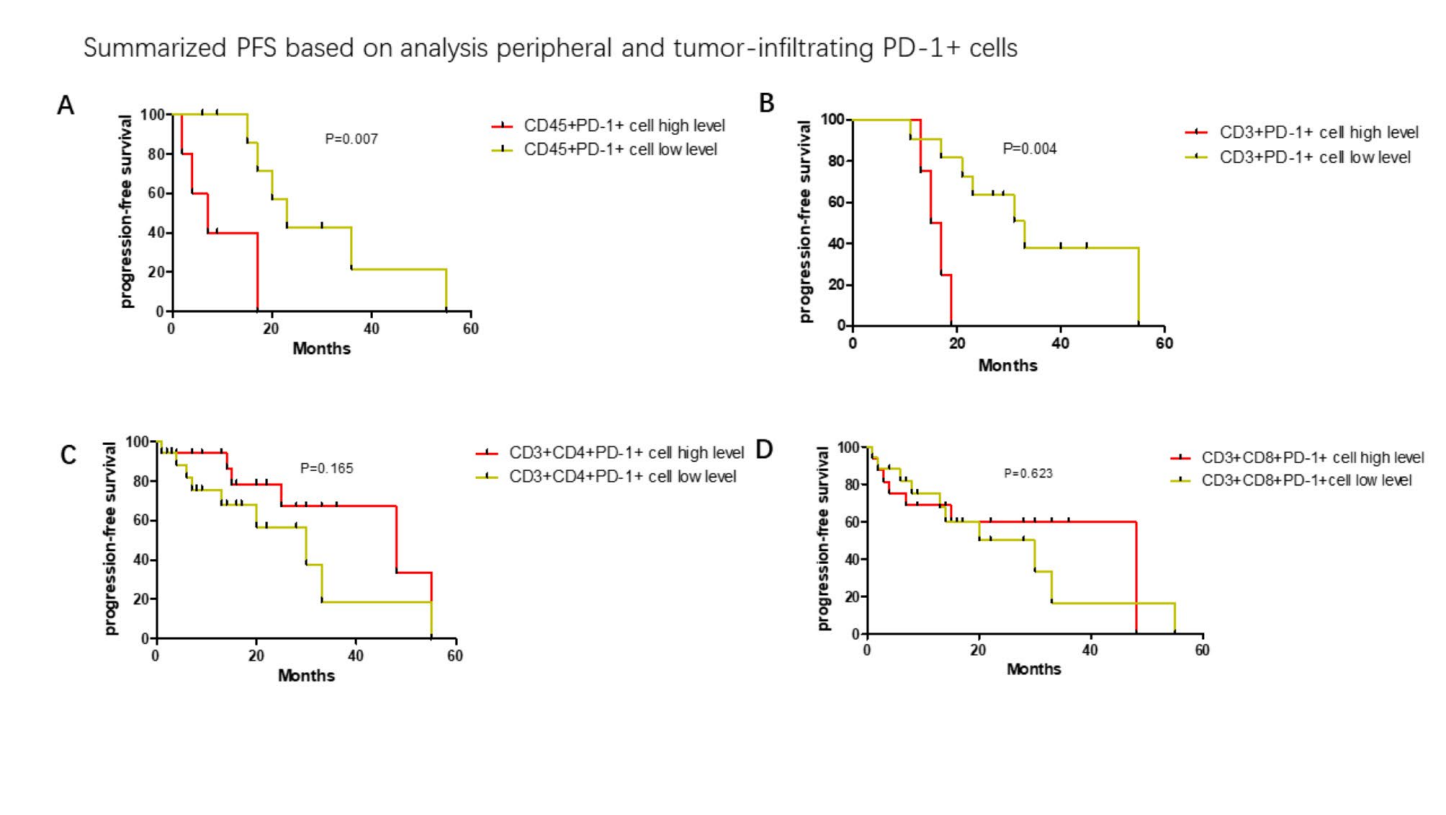
The progression-free survival (PFS) analysis of subgroups divided by levels of CD45 + PD-1 + cells, CD3 + PD-1 + T cells, CD3 + CD4 + PD-1 + T cells and CD3 + CD8 + PD-1 + T cells

**Fig. 8:**
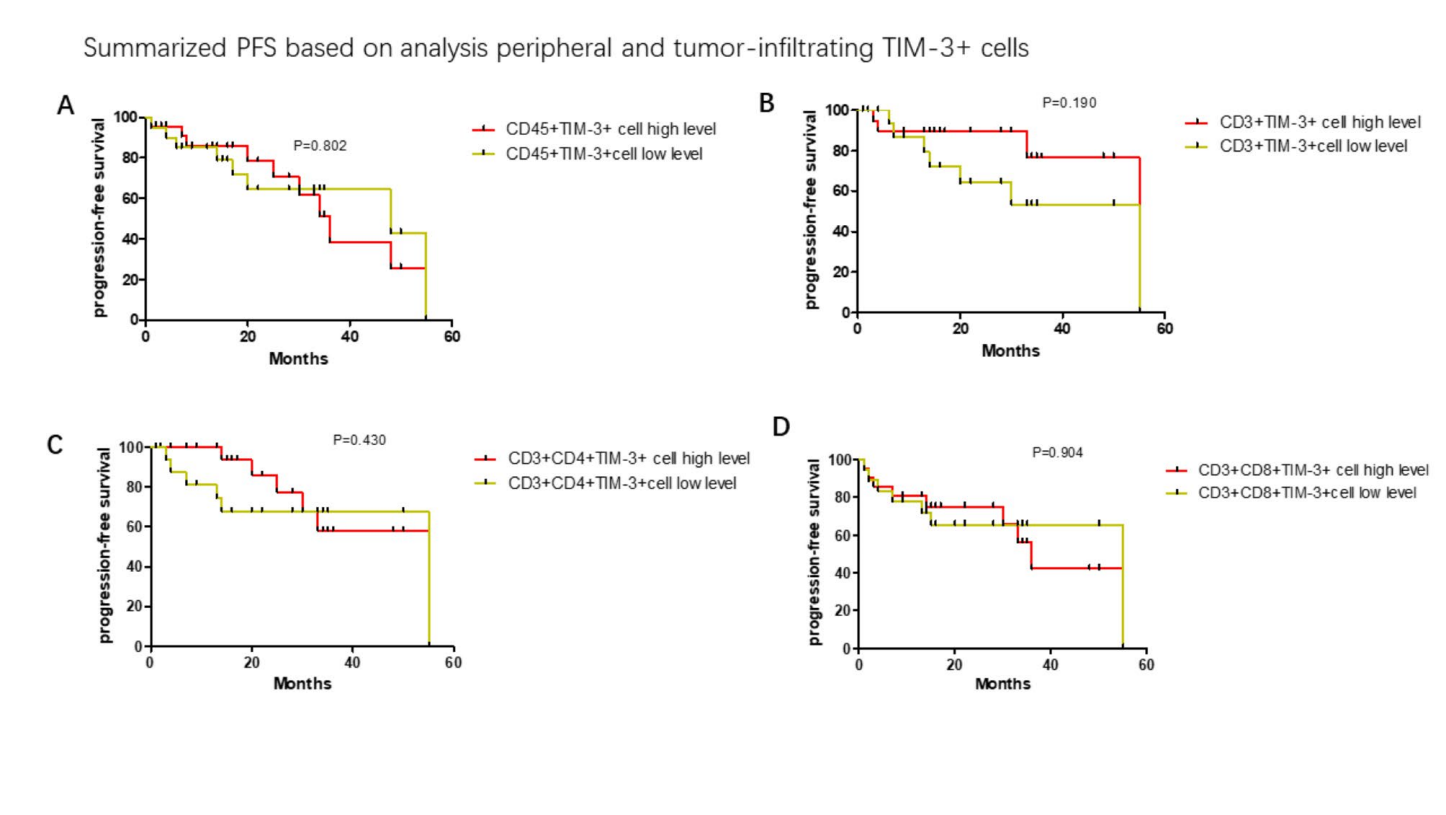
The progression-free survival (PFS) analysis of subgroups divided by levels of CD45 + Tim-3 + cells, CD3 + Tim-3 + cells, CD3 + CD4 + Tim-3 + T cells and CD3 + CD8 + Tim-3 + + T cells

The median percentages of PD-1 and Tim-3 on circulating T cells were calculated. The median frequencies were (CD45 + PD-1 + lymphocytes: 23.5%, CD3 + PD-1 + T cells:23.4%, CD3 + CD4 + PD-1 + T cells:31.1%, CD3 + CD8 + PD-1 + T cells: 31.9%, CD45 + Tim-3 + lymphocytes: 9.16%, CD3 + Tim-3 + T cells: 1.6%, CD3 + CD4 + Tim-3 + T cells: 1.5%, CD3 + CD8 + Tim-3 + T cells:1.8%). Progression-free survival was greater amongst those with lower levels of CD45 + PD-1 + lymphocytes (median PFS in high level:2.6 months vs. 8.3 months in low level, p = 0.007, Fig. 7A), CD3 + PD-1 + T cells (median PFS in high level:3.2 months vs13.1 months in low level, p = 0.004, Fig. 7B). There was non-significance effect ofCD3 + CD4 + PD-1 + T cells and CD3 + CD8 + PD-1 + T cells on the progression-free survival (p > 0.05, Fig. 7C and D). Unlike PD-1, percentages of Tim-3 exhibited on circulating T cells had no effect on PFS (p > 0.05, Fig. 8A and D).

## Discussion

PD-1 and Tim-3 were considered as the vital important immunosuppressive checkpoints in the immune escape and progression of cancer. Immune checkpoint blockade with anti-PD-1 antibodies had made great strides in the treatment of human malignancies. But some cancer, such as microsatellite-stable colorectal cancer, the success rate of immunotherapy was still low and not optimistic [17]. Preliminary data presented Tim-3 could act as an inhibitory receptor in different types of cancer, such as AML/MDS. Kuai et al. explored the co-expression of PD-1 and Tim-3 in stage I-III colorectal cancer tissue associated with poor prognosis of patients. The expression of PD-1 in colorectal cancer tissue associated with age, primary site, and lymphatic metastasis, and Tim-3 correlated with primary site [18]. Accumulated evidences proved PD-1 could act as the negative regulator in the immunity therapies of human osteosarcoma, lung cancer, renal cell carcinoma, and breast cancer [19–21]. PD-1 interacted with its ligands (PD-L1 or PD-L2) on the tumor cell surface, while Tim-3 mainly interacted with Galectin-9. In human body Tim-3, PD-1, Lag-3, and TIGIT w coregulated and co-expressed on CD4 + and CD8 + T cells [22, 23]. Riyao et al. had reported PD-1 physically interacted with Galectin-9 and TIM-3 to attenuate T cell apoptosis. Those findings revealed interesting crosstalk between immune checkpoint pathways in regulating T cell activity.[8]. The crosstalk between PD-1 and Tim-3 revealed their importance in regulating the immunosuppressive system and T cell activity. A recent study revealed that Tim-3 associated with adaptive resistance to the therapy of PD-1 immune blockade [24, 25]. However, whether there existed correlation between PD-1 and Tim-3 in peritoneal neoplasms patients’ peripheral blood or not, and whether there were links between the two immunosuppressive check points and primary sites & pathological types had not been explored.

Our study verified the assumption that within peritoneal neoplasms patients’ peripheral blood, PD-1 + and Tim-3 + on circulating T cells might participate in the cancer development. It was also found the peritoneal neoplasms patients presented with higher levels of circulating CD3 + CD4 + T cells, CD3 + CD8 + T cells, PD-1 + T lymphocytes, PD-1 + CD4 + T cells and PD-1 + CD8 + T cells, compared with health control. However, the differential percentage of CD3 + CD8 + PD-1 + Tim-3 + T cells was contrary to the above differences, CD3 + CD8 + PD-1 + Tim-3 + T cells presented higher level in normal control. That might be, human immune system was a complex compensatory system. Although CD3 + CD8 + PD-1 T cells were highly expressed in peritoneal tumors, circulating T lymphocytes were immunosuppressed in patients undergoing chemotherapy. Several studies showed the co-expression of PD-1 and Tim-3 indicated the several exhausted phenotype of T cells in proliferation and cytokine [26–28].

Most peritoneal neoplasms were malignant metastatic tumors of the abdomen, and some were primary tumors. Peritoneal neoplastic lesions normally be subdivided into primary peritoneal tumor and secondary peritoneal tumor. We divided the peritoneal neoplasms patients into primary and secondary groups, depending on whether the tumor had primary focus and limited to peritoneal tumor or not. Because of this, peritoneal neoplasms were more heterogeneous than other tumors. Our data showed the percentages of CD45 + PD-1 + lymphocytes, CD3 + PD-1 + T cells, and CD3 + CD4 + PD-1 + T cells were increased in the secondary peritoneal neoplasms group, compared with primary group, while Tim-3 had no statistical differences. But in the secondary group, CD45 + Tim-3 + lymphocytes, CD3 + Tim-3 + T cells, and CD3 + CD4 + Tim-3 + T cells correlated with the different primary sites, that suggested Tim-3 might be more relevant to the heterogeneity of organs. In the peripheral blood of different pathological groups, compared with Tim-3, percentages of PD-1 correlated with pathological types of peritoneal neoplasms. Higher percentages of circulating CD45 + PD-1 + lymphocytes and CD3 + PD-1 + T cells lymphocytes were found in adenocarcinoma group. Based on the following reports, the efficacy of immunotherapy was tightly correlated with the levels of PD-1 and Tim-3 in tumor infiltrating lymph nodes of the tumor immune microenvironment [29, 30]. Our data showed the assumption that those differences between PD-1 and Tim-3 might provide candidate immune biomarkers for peritoneal neoplasms patients’ personalized checkpoint directed therapy in the future. In this study, we also found patients with high frequencies of PD-1 + lymphocytes and PD-1 + T cells showed worse progression-free survival in multivariate analysis. That was consistent with the finding that expression of PD-1 + was associated with poor prognosis in human cancer.

Taken together, our results suggested peripheral PD-1 and Tim-3 expressions were associated with primary sites and pathological types of peritoneal neoplasms. This discover might provide a novel mechanism for clinical activity of peritoneal neoplasms immunotherapy. Of course, the present study had several limitations. Firstly, that might be required to for the further studies on the molecular mechanism of how PD-1 and Tim-3 mediated immunosuppression. Secondly, we analyzed the levels of PD-1 and Tim-3 using the method of flow cytometry, more methods such as immunohistochemistry and fluorescent quantitative PCR were needed, to investigate the deeper correlation between PD-1 and Tim-3 percentages and primary sites and pathological types of peritoneal neoplasms. Thirdly, this study was small sample size analyzed, we also need to enlarge the specimen to verify the cell location of PD-1 and Tim-3 on the circulating lymphocytes. In summary, patients with co-expression of PD-1 and Tim-3might have the guiding significance for diagnosis, treatment and prognosis of peritoneal neoplasms.

## Data Availability

All data generated or analyzed during this study are included in this published article. The datasets used and/or analyzed during the current study available from the corresponding author on reasonable request.
